# Bluster or Lustre: Can AI Improve Crops and Plant Health?

**DOI:** 10.3390/plants10122707

**Published:** 2021-12-09

**Authors:** Laura-Jayne Gardiner, Ritesh Krishna

**Affiliations:** IBM Research Europe, The Hartree Centre, Warrington WA4 4AD, UK; Ritesh.Krishna@uk.ibm.com

**Keywords:** AI, machine learning, crops, plant HEALTH, omics, disruptive technologies

## Abstract

In a changing climate where future food security is a growing concern, researchers are exploring new methods and technologies in the effort to meet ambitious crop yield targets. The application of Artificial Intelligence (AI) including Machine Learning (ML) methods in this area has been proposed as a potential mechanism to support this. This review explores current research in the area to convey the state-of-the-art as to how AI/ML have been used to advance research, gain insights, and generally enable progress in this area. We address the question—Can AI improve crops and plant health? We further discriminate the bluster from the lustre by identifying the key challenges that AI has been shown to address, balanced with the potential issues with its usage, and the key requisites for its success. Overall, we hope to raise awareness and, as a result, promote usage, of AI related approaches where they can have appropriate impact to improve practices in agricultural and plant sciences.

## 1. Introduction

Recent estimates predict that an increase of more than 60% in food will be needed by 2050 in order to feed the increasing global population [1]. These gains must be achieved while climate change threatens to reduce not only crop yields, but also their nutritious value through a range of mechanisms such as disease [2], drought [3], floods, heat, or low nutrient availability [4]. To keep up with the increase in the human population and with environmental changes, it is vital to increase crop yields. However, this goal is hampered by the limited availability of arable land and by the mounting pressure to reduce the use of agricultural chemicals such as fertilizers, herbicides, insecticides, and fungicides.

**Figure 1:**
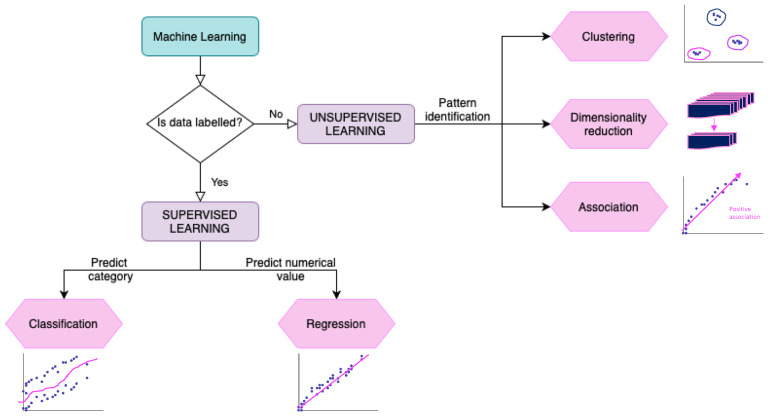
Summarizing different types of Machine Learning (ML) analyses. Exemplary analyses are broken down into supervised and unsupervised learning examples and the decision making involved in choosing these approaches.

Now, more than ever, it is critical to provide plant/crop breeders with new tools that will allow them to develop and sustain the next generation of crop varieties. These tools can come from a range of technologies, including but not limited to; methods to identify alleles linked to favourable traits, genetic marker combinations via high-throughput genotyping or *multi-omic* sequencing, gene editing, speed breeding strategies, yield prediction, monitoring and management of pests/disease, fertilization schemes and real-time crop surveillance e.g., through imaging and remote sensing. These technologies are producing data at an unprecedented rate and turning plant science into a data intensive discipline. Efficient computational analysis of these large volume datasets is essential for our understanding of crops and generation of new scientific insights to push the field forward. The application of artificial intelligence (AI) could be a powerful facilitator towards such goals, particularly when used in combination with the large volume of data collected through the above technologies. The terms Artificial Intelligence (AI) and Machine learning (ML) are commonly used interchangeably in the literature. However, AI incorporates the broader concept of simulating human intelligence by machines, for example, by learning or reasoning, while ML is a branch of AI which commonly involves computer learning in an autonomous fashion (Figure 1). Traditionally, we have witnessed multiple applications of supervised and unsupervised ML methods in plant science research. In supervised ML, the provision of labelled data enables the learning; algorithms are used to parse the data, learn from it, and then make a prediction about the labelling of new or previously unseen data. Unsupervised ML, on the other hand, involves discovering patterns in unlabeled data [5]. More recently, due to the availability of large, heterogenous and complex datasets, we are witnessing a rise in Deep learning (DL) based methods that use neural networks to simulate human decision-making [6] and may include supervised, semi-supervised and unsupervised ML methods. In this review, we will use the umbrella term of AI to cover both AI and ML for the sake of better readability.

The aim of this review is to provide the reader an overview of how AI algorithms are being applied to advance our understanding of plant health, and to highlight how they could be used to aid food security. In doing so we highlight the challenges in the field that AI can address. As such we begin with a focus on AI for the usage and interpretation of a range of high-throughput phenotyping datasets e.g., image-based. Then we investigate analyses that involve the integration of state-of-the-art, data rich technologies, such as genotype-to-phenotype analysis using *multi-omic* sequencing datasets. Throughout we consider the challenges and problem areas where AI methods can be improved. Finally, we detail emerging areas of interest in the field which can be led by and the potential issues with the usage of AI approaches e.g., the need for transparency and interpretability of approaches. We hope to increase the recognition, as well as the accessibility of AI tools in agricultural and plant science research more generally. From our investigations we propose that a significant degree of bluster with regard to the usage of AI may be due to its increasing usage where the benefits of AI over current practice is not made clear or not comprehensively tested. We suggest that to discriminate the bluster from the lustre it is essential to highlight advances that have been made to current practice with adequate comparisons to existing statistical models, existing approaches, or otherwise.

## 2. AI in Plants

### 2.1. Phenotyping

The collection of plant phenotypic information can be a manual and time-consuming process, potentially requiring significant domain expertise. This collection process limits the quantity of available phenotypic data for a given plant species and the purpose of its usage, like facilitating genotype-to-phenotype analysis. The quality of phenotypic data can vary according to the collection methodology and the degree of induced human error. To accelerate discovery and increase reproducibility in this area, high-throughput plant phenotyping is an area of active development to automate retrieval of plant phenotypes. One example of this is the usage of sensors to record environmental (water intake, light, temperature, humidity) and physical (plant weight, height etc.) measurements of interest that can be incorporated in ML models, to depict phenotype and phenotype-environmental interactions [7]. Another example is image-based phenotyping that gives increased power to evaluate morphological features. As a result, imaging is increasingly being seen as the gold-standard for plant phenotyping, having the capability to also enable real-time analytics and monitoring.

Image-based phenotyping, e.g., advanced microscopy and field/plot drone imaging [8], has the caveat that it can generate huge datasets that require time-consuming, expert, manual interpretation to extract specific phenotypic measurements from. These extracted phenotypic measurements can then be associated with genotype or otherwise. In fact, it is proposed that processing and extracting features from images could be the new phenotyping bottleneck [9], and that this is an area of interest where AI could provide automated solutions. For example, AI was successfully applied to detect different disease development stages of powdery mildew in squash using both lab-based, and unmanned aerial vehicle (UAV) field-based, hyperspectral imaging [10]. We have seen great advancements in deep learning-based methods like convolutional neural networks (CNN), that have been used with great degree of success in many fields, including plant sciences, for image-based ML goals. When we focus on pest/disease detection and monitoring, Kawasaki et al. [11] used a CNN to classify cucumbers as diseased or non-diseased from leaf imaging, identifying regions of interest for disease diagnosis. Similarly CNNs were used by Fuentes et al. [12] alongside images detected in-place by camera devices, to allow real-time detection of diseases and pest in tomato plants. However, CNNs are not the only means for image analysis, for example, Jeon et al. [13] developed an image processing algorithm for discriminating individual weed and crop plants from robot captured field images using an Artificial Neural Network (ANN). Classical ML approaches like Random Forests were also used by Gao et al. [14] for accurate weed detection in early season maize fields from UAV imagery.

In line with this work on disease detection, it is acknowledged that great value can be achieved via the assessment of other stresses beyond diseases. One such widely studied example is plant water stress estimation. An et al. [15] classified water stress in maize based on optimum moisture, light drought, and moderate drought stress. To do this they used the pre-trained CNNs Resnet50 and Resnet120 and digital camera images. Various studies followed this work, each aiming to improve the demonstrated performance and accuracy [16,17]. Whereas, Chandel et al. [18] compared three deep learning models (AlexNet, GoogLeNet and Inception V3) for the identification of water stress in maize, okra and soybean crops from images. In this analysis GoogLeNet showed superior performance when accuracy was assessed.

Many of the detailed approaches do not explicitly consider temporal features, for example those from sequential images or videos. Li et al. [19] tackled this problem using BiLSTM networks to extract features from sequential digital images of maize and sorghum and to successfully identify plant water stress, even at an early stage.

The monitoring and detection of disease and plant stress is important not only for the determination of crop management strategies e.g., pesticide application, but also for yield prediction. There is a large body of research that focuses on yield prediction and forecasting directly, and there are a growing number of examples of such predictions from image-based analysis. You et al. [20] applied CNNs and RNNs for soybean crop yield prediction that was based on a sequence of remotely sensed multi-spectral satellite images enabling them to include information on vegetation growth and thus on agricultural outcomes. Garcia-Martinez et al. [21] analysed different multispectral and red-green-blue (RGB) vegetation indices, as well as the digitally estimated canopy cover and plant density, in order to estimate corn grain yield using a neural network model. Yang et al. [22] investigated the ability of CNN to estimate rice grain yield using remotely sensed UAV images and proposed a CNN model that provided robust yield forecast throughout the ripening stage. Finally, Khaki et al. [23] developed a deep learning framework, comprised of a hybrid CNN-RNN model, to predict crop yield based on environmental data and management practices. Such CNNs are capable of processing data with formats such as one-dimensional data (signals and sequences), two-dimensional data (images), and three-dimensional data (videos).

AI has been applied for image-based plant phenotyping to identify more general developmental plant phenotypes. For example Ubbens et al. [24] used CNNs for plant leaf counting and age regression for images on plant rosettes. While Pound et al. [25] used CNNs to count spike and spikelets in wheat images in order to study plant development. Falk et al. [26] integrated time series image capture with computer vision and a ML based segmentation approach to image soybean roots and study traits including shape, length, number mass and angle. Taghavi Namin et al. [27] proposed the usage of an alternate deep learning technique—Recurrent Neural networks (RNNs) and in particular Long Short-Term Memories (LSTMs), to model temporal growth patterns due to their ability to learn long-range dynamics.

Many of these AI models learn in a supervised fashion from labelled datasets, and are limited by the availability and quality of training examples. An alternate approach is to include the usage of semi-supervised or unsupervised methods that are not dependent on availability of curated labelled datasets. For example, Al-Shakarji et al. [28] developed an unsupervised learning method for leaf detection, extraction and counting in *Arabidopsis.* Semi-supervised approaches such as transfer learning can also be used where a neural network can be trained on a more general dataset and then later fine-tuned on a specific dataset of interest. This offers the advantage that the specific dataset of interest can be much smaller by comparison. Douarre et al. [29] used this technique to transfer learning from synthetic or simulated data to allow soil-root segmentation in X-Ray Tomography images.

The examples given here for the usage of AI demonstrate its lustre since they typically yield a clear advantage over the existing methods or practices for the same task. To highlight this and instil confidence in their offerings, many studies offer detailed comparisons with the traditional approaches, and highlight the benefits over what otherwise would have been an arduous manual task e.g., visually inspecting plants for appearance of disease, or, to define developmental stages. The examples that we have covered included a range of both controlled experimental conditions e.g., laboratory or greenhouse-based, and field applications. One could argue that highly accurate performance of AI in a laboratory setting, with no demonstratable translation to the field, could contribute to the bluster surrounding the application of AI for certain tasks. If we take disease detection as an example, there are certainly additional considerations that the above cited authors have made when they move from the laboratory to field settings for monitoring of disease, these include noise introduced by poorer image quality because of the imaging techniques used (e.g., UAV, satellite, or robot capture images) and weather effects etc. As a result, many of the field-based solutions we see have been developed specifically with these considerations in mind. However, for the correct use-case it is important to recognise the value of laboratory-based approaches e.g., if the disease detection is part of a controlled laboratory experiment to identify the genes underlying key traits.

### 2.2. Genotype-to-Phenotype

Linking alleles or genomic regions to traits of interest i.e., genotype-to-phenotype, is one of the fundamental challenges in biology. This enables the derivation of desirable genetic markers that underly favorable phenotypes and that are therefore targets to be combined when breeding. To date much work in plants in this area uses traditional statistical approaches such as QTL mapping using experimental populations [30] or Genome Wide Association Studies (GWAS) where diverse panels are available [31]. Although, aligned to this is the ability to predict complex traits (where typically many small-effect alleles contribute) using a large selection of genome-wide genetic markers (referred to as Genomic Prediction [32]), which is a major research goal that has the potential to accelerate crop breeding and where AI is making an impact. Initial work by Meuwissen et al. [32] proposed a selection of statistical methods for Genomic Prediction including ridge regression Best Linear Unbiased Prediction (rrBLUP) and Bayesian approaches. While others have demonstrated the utility of other approaches e.g., Least Absolute Angle and Selection Operator (LASSO) based approaches [33,34,35], genomic BLUP (GBLUP) and elastic nets (EN) [36]. Most of the earlier statistical methods that have been used for Genomic Prediction are linear in their mapping of genotype-to-phenotype. However, non-linear methods have been proposed for Genomic Prediction to better represent the biological interactions of complex traits which may include phenomenon such as epistasis. Such non-linear methods include ML based methods like decision tree algorithms and deep learning that have the added advantage of scaling easily to enable analysis of large datasets. We will highlight some exemplars of the application of ML for Genomic Prediction and comparisons (where available) with other classical approaches.

Firstly, Holliday et al. [37] used a Random Forest to identify optimized combinations of SNPs to predict adaptive phenotypes in the widespread conifer Sitka spruce (*Picea sitchensis*). They quantified the strength and direction of pairwise interactions between SNPs to evaluate the role of epistasis in shaping these phenotypes and demonstrate the power of Random Forest to identify subsets of markers that are most important to climatic adaptation. Secondly, Long et al. [38] predicted grain yield in wheat from dense molecular markers. They showed that using support vector regression (SVR) with a Gaussian radial basis function (RBF) kernel outperformed a linear kernel and Bayesian Lasso, and noted the benefit of a non-linear kernel when the phenotype to be predicted had a non-linear dependency on genotypes. Thirdly, González-Camacho et al. [39] compared linear Bayesian LASSO regression with two non-linear regression models, reproducing kernel Hilbert spaces (RKHS) regression and RBF neural networks (RBFNN) on maize genotyping data and evaluated for several trait–environment combinations. They observed a slight and consistent superiority of RKHS and RBFNN over the additive Bayesian LASSO model where the RKHS and RBFNN models also captured epistatic effects. Next, González-Camacho et al. [40] compared the performance of a multi-layer perceptron (MLP) classifier versus a probabilistic neural network (PNN) using single nucleotide polymorphism (SNP) information to select maize and wheat individuals of a specific phenotypic class. They found the PNN to be more accurate than MLP for assigning lines to the correct class for the analysed complex traits. Ma et al. [41] developed a deep learning method, named DeepGS, to predict phenotypes from high-dimensional genotypic data using a deep CNN. They proposed the use of DeepGS as a complementary method to the commonly used RR-BLUP as an ensemble learning approach for more accurately selecting individuals with high phenotypic values.

When we next focus on work that has been published more recently, this typically yields conclusions regarding the utility of AI that are also in line with previous work. For example, Sousa et al. [42] compare a range of ML, DL and traditional statistical approaches to predict the genetic resistance of Arabica coffee to orange leaf rust. They input genotypic markers into their models and used accuracy and apparent error rate (APER) to compare an ANN, a Decision Tree ML approach with Generalized Bayesian Lasso (GBLASSO). Overall, the authors found that the ML-based methodologies outperformed GBLASSO showing higher accuracies. However, when they looked at APER the Decision Tree produced higher errors (24.9%) than the GBLASSO (22.7%) due to high variance in terms of prediction, this remained true until they tested usage of ensemble methods such as bagging, random forest and boosting that combine multiple Decision trees to reduce variability (reducing error to a minimum of 19.5%). In another example, Zingaretti et al. [43] compared and evaluated the predictive accuracy of linear and DL techniques using genotyping data from strawberries and blueberries to predict agronomic traits including yield, weight and fruit size. In terms of genomic prediction, linear Bayesian models were better than the DL method that they used (CNNs) for the full additive architecture, whereas the opposite was observed under strong epistasis i.e., dependent on the use-case. After extensive testing the authors noted that by using a parameterization capable of considering these non-linear effects, Bayesian linear models could match or even exceed the predictive accuracy of DL. Finally, the work presented by Sandhu et al. [44] evaluated the potential of DL models for the prediction of traits such as grain yield, grain protein content, heading date, plant height, and test weight using SNPs from the Washington State University spring wheat breeding program. They compared the performance of two DL algorithms, namely multilayer perceptron (MLP) and a CNN, with a more traditional statistic approach in the form of ridge regression best linear unbiased predictor (rrBLUP). The found that the DL models gave 0 to 5% higher prediction accuracy than rrBLUP model for all five traits with, MLP producing a 5% higher prediction accuracy than CNN for grain yield and grain protein content.

We previously proposed that some of the bluster surrounding AI can be attributed to the assumption of its superiority over current methods without detailed comparisons. It has been observed in other studies that different algorithms may produce different results, these studies often highlight the fact that no one single method performs the best in all cases, even when comparing only amongst AI approaches. Azodi et al. [45] highlighted the importance of comparing algorithms across a diverse range of datasets. This work used data of 18 traits across six plant species with different marker densities and training population sizes, and the authors compared the performance of six linear and six non-linear algorithms. The authors reported that no one algorithm performed best in all cases, but that those predictions based on a combination of results from multiple algorithms (i.e., ensemble predictions) performed consistently well. Similar comparative studies were carried out by Spindel et al. [46] for rice, where different methods performed better for different datasets e.g., to predict plant height, a Random Forest produced the most accurate models.

### 2.3. Omic Data

Many of the highlighted studies that use AI for Genomic Prediction, or to connect genotype-to-phenotype generally, incorporate genotypic markers as SNPs. In many cases these have been derived from array-based technologies. SNP arrays are a high-throughput, relatively cost-efficient genotyping assay that can typically be automated in the laboratory (experiment and analyses). They have been widely used to genotype crops (see the review by You et al. [47] for further details), where even for complex polyploid plants such as wheat and strawberry there are SNP arrays available holding up to 820 K SNPs. However, as the cost of sequencing technologies decreases there is an increasing desire to adopt *omic* technologies such as genomics (sequencing the DNA of the genome), transcriptomics (sequencing RNA to determine which genes expressed by the organism at any one time), proteomics (large-scale study of proteins that are produced by an organism) and the epigenome (chemical modifications to DNA e.g., methylation of cytosines, that can turn genes on or off). Individually within their respective layers, or more powerfully in combination (*multi-omics*), these technologies have the capability to capture more of the whole picture of what is happening inside the cell rather than, for example, using a subset of SNPs on an array alone.

AI is playing an increasingly important role for exploration of the genome and its complexities and to assess the interaction between *omic* layers. For example, Korani et al. [48] compared a range of ML algorithms (including a neural network, logistic regression, K-nearest neighbors and Decision trees) to discriminate true positive and false positive SNPs in polyploid peanut plants. They achieved high accuracy directly from both peanut RNA-seq and whole-genome shotgun (WGS) resequencing data. The review by Mochida et al. [49] discusses the large range of ML-based methods derived that typically use transcriptome data to construct gene regulatory networks (GRNs). The GRNs represent gene interactions with each other and with other cell substances to direct the expression of mRNA and proteins and ultimately determine the function of the cell. For example, Mochida et al. [49] highlight GENIE3 [50], a tree-based ML algorithm that has been widely used to infer GRNs from transcriptomic (RNA-seq) data in a range of species. Furthermore, there is a growing body of research that aims to use deep learning to predict transcriptomic information directly from DNA sequence or epigenetic marks typically using representations of transcription factor binding sites (TFBS) [51,52], enhancers [53], histone modifications [54], open chromatin regions [55], or promoters [56]. Similarly, Gardiner et al. [57] incorporated both promoter and transcript sequence as k-mer profiles to represent *de novo* identified TF, miRNA and RNA binding sites, to predict complex dynamic transcriptomic profiles using ML-based models.

#### 2.3.1. Challenges from Omic Data

*Omic* data generally originates as large volumes of raw sequencing reads that require downstream processing via bioinformatic workflows to derive high quality features. Genomic, transcriptomic and epigenomic data can commonly be processed to derive features such as SNPs, gene expression counts and cytosine methylation (%) respectively. It is essential that bioinformatics workflows are carefully curated and rigorously tested in order to process the raw sequencing data into high quality and valid features, and therefore, bioinformatics acts as a huge foundational research area. Bioinformatically processed features can have high dimensionality, they can be sparse and can also be inherently noisy, showing variation between replicates and experimental batch effects. Some of these shortcomings can be overcome by computationally efficient ML–based algorithms that make minimal assumptions about the data’s probability distributions and generation and provide a suitable platform for computational inference.

**Figure 2:**
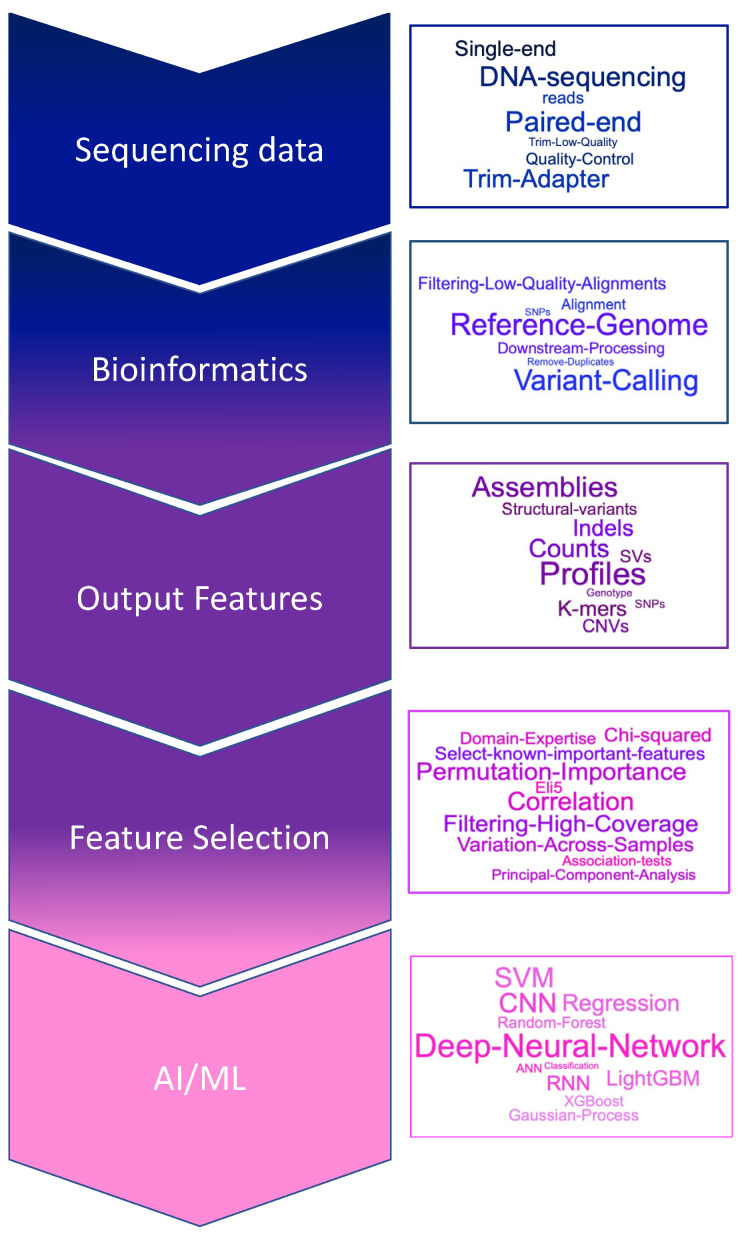
Flow chart of the processing pipeline from raw *omic* sequencing data to ML-analysis. Pipeline uses genomic DNA sequencing data as an exemplar to show a typical bioinformatic processing pipeline components, Quality Control (QC) steps to ensure high quality features are generated, a selection of potential output features (dependent on the analysis aim), feature selection techniques to reduce the dimensionality of the resultant feature sets and finally a range of possible AI/ML methods.

The high dimensionality of *omic* datasets means that for the features derived from the raw data, there are typically more of them (or a large number) compared to the available number of samples. In fact, *omic* studies can generate huge numbers of input features for ML models and therefore feature selection and bioinformatic filtering approaches can be used to reduce these numbers to select the most relevant features for downstream analysis. For example, cases of high dimensionality are highlighted by studies in wheat, generating evidence of gene expression for >83 K genes from 209 RNA-seq samples [58], while in another study there were >716 K SNPs and methylation information for >850 K cytosines per sample across 104 samples from genomic and epigenomic sequencing [59,60]. These examples also highlight data sparsity where, for example, for the >716 K SNPs only 53 K were observed with what the authors deemed a sufficient depth of sequencing coverage (5X) in more than 1 sample i.e., many identified features could be unique to one sample or to small sample subsets. Filtering features that show such sparsity (and filtering generally), could risk losing some information from downstream models that may be important when models are applied to new independent datasets. But feature filtering, with strict bioinformatic processing, can reduce not only dimensionality issues but also reduce noise in the *omic* datasets as it is possible that features unique to e.g., a single sample, could be errors or else largely uninformative for modelling global trends. Figure 2 uses genomic data as an exemplar to summarize the processing of *omic* data, its features and suggested potential solutions to reduce dimension and complexity of these feature sets prior to ML analyses.

The removal of low quality or sparse features addresses some of the issues common to *omic* data. However, even in a high quality, validated feature set e.g., a set of validated SNPs, there could be both relevant as well as redundant features present in the data. Therefore, feature selection can be employed (Figure 2) where, for example, a univariate filtering approach can be used to rank features and filter out the least promising before downstream modelling, this can not only reduce the dimensionality of the datasets to improve the performance of the ML model (reducing CPU-time memory and likelihood of overfitting), but can also provide insight into the underlying biological processes represented by the data i.e., answering the question-are there features that are irrelevant and/or redundant for the biological study being conducted? Common feature selection methods (that are independent of the final learning model) include association tests (e.g., chi-squared) or correlation-based filters. These have the caveat that they could remove relevant features that are meaningless when considered in isolation but that can be useful in combination. This affect can be mitigated using algorithms such as Principal Component Analysis (PCA) where new smaller sets of orthogonal features (principal components) are obtained by maximizing the variation of the original features, to visualize the data in a lower-dimensional space. Though further processing is required for a domain specialist to interpret these new features. The work by Perez-Riverol et al. [61] compares feature selection approaches, deriving a feature selection workflow for high-dimensional *omic* data.

In addition to the more commonly used feature selection methods in Figure 2, ML-based methods have been employed directly to prioritize features that can be used as input into GP analyses. For example, Li et al. [62] used Random Forests, Gradient Boosting Machine (GBM) and XgBoost to identify the top ranked SNPs to construct genomic relationship matrices for the estimation of genomic breeding values (GEBVs). They found that the performance of the SNP subsets selected by RF and GBM was better than that of other sampling strategies and comparable to using the whole SNP panel, and they identified SNPs with direct links to candidate genes affecting growth. In contrast, the work by Degenhardt et al. [63] investigated downstream methods that were entwined with the final learning model and looked to define a minimal set of variables needed for the best predictions. They compared a range of methods that perform recursive feature elimination (RFE) where prediction error after removal of a feature was used to determine its predictive value (permutation importance). In the case of the availability of enough data samples, one may also use deep learning-based methods, opposed to classical methods explained above, in order to let the algorithm automatically curate features from the data, and thus, remove the need for an explicit feature curation and selection stage. These deep learning methods also do not need to hold all the data in memory, like many of the classical algorithms, making them more computationally efficient, and they have a different way of handling the problem caused by the dimensionality of the data.

#### 2.3.2. Omic Data Integration

As researchers strive to gain a fuller picture of a biological system by integrating multiple *omic* layers (*multi-omic*), it will become critical to represent the complex interactions, not only within but between these layers, to derive meaningful insights. Methods have been proposed for this including using multi-layer networks and Graph Convolutional Networks (GCN). The work by Haas et al. [64] discusses the usage of multi-layer networks to represent all the functional interactions within and across all the *omic* layers of the cell for a given organism. However, they suggest that this is currently limited by incomplete *omic* data and propose the usage of ML algorithms to fill the gaps in our knowledge of how the information flows across the *omic* layers e.g., as described by Vert et al. in [65]. Alternatively, the work by Wang et al. [66] proposed the usage of *Multi-Omics* gRaph cOnvolutional NETworks (MORONET) for biomedical classification. MORONET incorporates mRNA expression data, DNA methylation data, and miRNA expression data to firstly employ *omics*-specific learning and then *cross-omics* correlation learning for *multi-omics* data classification. Specifically, MORONET utilizes Graph Convolutional Networks (GCNs) that can take advantage of both the *omics* features and the correlations among patients described by the patient similarity networks. Such work has largely been reported in the domain of biomedical research, and extensive translation of the learning into plants currently does not appear to be widespread.

The adoption of *multi-omic* data in plant science research has resulted in availability of a large volume of curated and non-curated datasets. As such, we are witnessing a rise in the development and usage of Knowledge Base technologies, in particular Graph Databases [67,68], for organizing the knowledge captured by the scientific community. Graph databases capture the interconnected nature of the biology and are proving to be a valuable technology for organizing *omic* data and applying graph-theoretic and NLP based AI techniques to answer questions such as “what-if”, to identify causal relationships, and to perform gap analysis. Dai et al. proposed HRGRN [69], which is an example of Graph powered database for *Arabidopsis* signaling transduction, metabolism and gene regulatory networks. While Venkatesan et al. [70] developed Agronomic Linked Data (AgroLD), a knowledge-based system that uses Semantic Web technologies and standard ontologies to integrate and query data for various plant species, including corn, rice and wheat. KnetMiner [71] represents another plant specific and extensive knowledge base that was developed with the aim to accelerate the gene-trait discovery process. Graph databases are emerging as an important technical area in the plant science domain, and we expect the adoption of such databases within the plant science community to continue to grow.

## 3. Emerging Areas of Interest in the Field

### Explainability and Interpretability

AI models can sometimes be referred to as ‘black boxes’, this phrasing is used because in many cases the inner logic of the model cannot be easily understood by humans. Such a scenario is thought to be a potential barrier to the uptake of AI in practical applications, adding to the bluster behind such approaches, where the user could feel unsure or doubtful about acting on a model’s prediction if they cannot see the reasoning behind it. Graph databases attempt to answer such questions to an extent. Parallelly, Explainable AI algorithms are proposed as methods to illuminate what is inside the ‘black box’ i.e., they help us to understand and interpret why predictions have been made. Clarity as to how a prediction was derived, for example identifying important patterns and/or features that underly an AI model, could increase the level of trust from the end-user e.g., a breeder, as described by Harfouche et al. [72]. Additionally, model explanation was proposed by Gardiner et al. [57] as a method for hypothesis generation to identify, rank and therefore prioritize features that could drive the phenotype being predicted. The authors suggest that this could allow a user to streamline the features selected for downstream experimental investigation and validation. The study by Ghosal et al. [73] in soybean used explainable deep machine learning to automate plant stress identification, delivering trained pathologist-level performance and using explanation to identify which visual plant stress symptoms are important to make predictions. Finally, Newman et al. [74] trained an ensemble of ML models combining crop weather, ground-sensor, soil, chemical and fertiliser dosage, management, and satellite data, to produce robust cross-continent yield models. They provided explanation of these models to reveal fundamental drivers of crop behaviour and complex interactions predicting yield and agronomic traits.

## 4. Discussion

In this review we have explored the usage and potential advantages of AI across a variety of applications relevant to the agricultural and plant sciences. Firstly, we looked at the lucrative potential for AI to overcome a phenotyping bottleneck (manual curation or human-led visual inspection of phenotypes) by automating the processing and extraction of features from phenotyping analyses e.g., via imaging or videos. Secondly, we reviewed current work on Genomic Prediction where the consensus was that no one algorithm performed best in all cases, but that those predictions based on a combination of results from multiple algorithms typically performed best. Here, we noted the importance of detailed comparisons between AI approaches and existing classic statistical methods to prove an advantage can be gained—and thus, not amplify the bluster surrounding AI, which we propose can partly be attributed to the assumption of AI’s superiority over existing methods without detailed comparisons. Next, we highlight the challenges encountered with *multi-omic* datasets e.g., high-dimensionality, followed by discussing the benefits of graph-based data integration and interpretable predictions, all of which are beginning to be addressed in the AI domain and are the subject of active development in the field. We conclude that, dependent on the question being asked and the availability of suitable data, AI has great potential to improve crops and plant health. The biggest advantages of AI could be yet to come as we continue to develop AI-based approaches that more fully capture the biological complexity of the systems under analysis e.g., via graph-based methods, and open the door for the next level of research advancement, insight generation and progress that we must harness to develop the next generation of crops.

## Data Availability

Not applicable.

## References

[1] Grafton R.Q., Daugbjerg C., Qureshi M.E. (2015). Towards food security by 2050. Food Secur..

[2] Corredor-Moreno P., Saunders D.G.O. (2019). Expecting the unexpected: Factors influencing the emergence of fungal and oomycete plant pathogens. New Phytol..

[3] Li Y., Ye W., Wang M., Yan X. (2009). Climate change and drought: A risk assessment of crop-yield impacts. Clim. Res..

[4] Mall R., Gupta A., Sonkar G. (2017). Effect of Climate Change on Agricultural Crops. Current Developments in Biotechnology and Bioengineering.

[5] Reese H. (2017). Understanding the Differences between AI, Machine Learning, and Deep Learning. https://deeplearning.lipingyang.org/wp-content/uploads/2016/11/Understanding-the-differences-between-AI-machine-learning-and-deep-learning-TechRepublic.pdf.

[6] Wang H., Cimen E., Singh N., Buckler E. (2020). Deep learning for plant genomics and crop improvement. Curr. Opin. Plant Biol..

[7] Fahlgren N., Gehan M.A., Baxter I. (2015). Lights, camera, action: High-throughput plant phenotyping is ready for a close-up. Curr. Opin. Plant Biol..

[8] Tsouros D.C., Bibi S., Sarigiannidis P.G. (2019). A review on UAV-based applications for precision agriculture. Information.

[9] Tsaftaris S.A., Minervini M., Scharr H. (2016). Machine Learning for Plant Phenotyping Needs Image Processing. Trends Plant Sci..

[10] Abdulridha J., Ampatzidis Y., Roberts P., Kakarla S.C. (2020). Detecting powdery mildew disease in squash at different stages using UAV-based hyperspectral imaging and artificial intelligence. Biosyst. Eng..

[11] Kawasaki Y., Uga H., Kagiwada S., Iyatomi H., Bebis G., Boyle R., Parvin B., Koracin D., Pavlidis I., Feris R., McGraw T., Elendt M., Kopper R., Ragan E. (2015). Basic Study of Automated Diagnosis of Viral Plant Diseases Using Convolutional Neural Networks. Advances in Visual Computing.

[12] Fuentes A., Yoon S., Kim S.C., Park D.S. (2017). A Robust Deep-Learning-Based Detector for Real-Time Tomato Plant Diseases and Pests Recognition. Sensors.

[13] Jeon H.Y., Tian L.F., Zhu H. (2011). Robust Crop and Weed Segmentation under Uncontrolled Outdoor Illumination. Sensors.

[14] Gao J., Liao W., Nuyttens D., Lootens P., Vangeyte J., Pižurica A., He Y., Pieters J.G. (2018). Fusion of pixel and object-based features for weed mapping using unmanned aerial vehicle imagery. Int. J. Appl. Earth Obs. Geoinf..

[15] An J., Li W., Li M., Cui S., Yue H. (2019). Identification and Classification of Maize Drought Stress Using Deep Convolutional Neural Network. Symmetry.

[16] Jiang B., Wang P., Zhuang S., Li M., Gong Z. Drought Stress Detection in the Middle Growth Stage of Maize Based on Gabor Filter and Deep Learning. Proceedings of the 2019 Chinese Control Conference (CCC).

[17] Zhuang S., Wang P., Jiang B., Li M. (2020). Learned features of leaf phenotype to monitor maize water status in the fields. Comput. Electron. Agric..

[18] Chandel N.S., Chakraborty S.K., Rajwade Y.A., Dubey K., Tiwari M.K., Jat D. (2021). Identifying crop water stress using deep learning models. Neural Comput. Appl..

[19] Li H., Yin Z., Manley P., Burken J.G., Shakoor, Fahlgren N., Mockler T. (2018). Early Drought Plant Stress Detection with Bi-Directional Long-Term Memory Networks. Photogramm. Eng. Remote Sens..

[20] You J., Li X., Low M., Lobell D., Ermon S. Deep Gaussian Process for Crop Yield Prediction Based on Remote Sensing Data. Proceedings of the Thirty-First AAAI Conference on Artificial Intelligence.

[21] García-Martínez H., Flores-Magdaleno H., Ascencio-Hernández R., Khalil-Gardezi A., Tijerina-Chávez L., Mancilla-Villa O., Vázquez-Peña M. (2020). Corn Grain Yield Estimation from Vegetation Indices, Canopy Cover, Plant Density, and a Neural Network Using Multispectral and RGB Images Acquired with Unmanned Aerial Vehicles. Agriculture.

[22] Yang Q., Shi L., Han J., Zha Y., Zhu P. (2019). Deep convolutional neural networks for rice grain yield estimation at the ripening stage using UAV-based remotely sensed images. Field Crop. Res..

[23] Khaki S., Wang L., Archontoulis S.V. (2020). A CNN-RNN Framework for Crop Yield Prediction. Front. Plant Sci..

[24] Ubbens J.R., Stavness I. (2017). Deep Plant Phenomics: A Deep Learning Platform for Complex Plant Phenotyping Tasks. Front. Plant Sci..

[25] Pound M.P., Atkinson J.A., Wells D.M., Pridmore T.P., French A.P. Deep Learning for Multi-task Plant Phenotyping. Proceedings of the 2017 IEEE International Conference on Computer Vision Workshops (ICCVW).

[26] Falk K.G., Jubery T.Z., Mirnezami S.V., Parmley K.A., Sarkar S., Singh A., Ganapathysubramanian B., Singh A.K. (2020). Computer vision and machine learning enabled soybean root phenotyping pipeline. Plant Methods.

[27] Namin S.T., Esmaeilzadeh M., Najafi M., Brown T.B., Borevitz J.O. (2018). Deep phenotyping: Deep learning for temporal phenotype/genotype classification. Plant Methods.

[28] Al-Shakarji N.M., Kassim Y.M., Palaniappan K. Unsupervised Learning Method for Plant and Leaf Segmentation. Proceedings of the 2017 IEEE Applied Imagery Pattern Recognition Workshop (AIPR).

[29] Douarre C., Schielein R., Frindel C., Gerth S., Rousseau D. (2018). Transfer Learning from Synthetic Data Applied to Soil–Root Segmentation in X-Ray Tomography Images. J. Imaging.

[30] Gardiner L.-J., Bansept-Basler P., El-Soda M., Hall A., O’Sullivan D.M. (2020). A framework for gene mapping in wheat demonstrated using the Yr7 yellow rust resistance gene. PLoS ONE.

[31] Joynson R., Molero G., Coombes B., Gardiner L., Rivera-Amado C., Piñera-Chávez F.J., Evans J.R., Furbank R.T., Reynolds M.P., Hall A. (2021). Uncovering candidate genes involved in photosynthetic capacity using unexplored genetic variation in Spring Wheat. Plant Biotechnol. J..

[32] Meuwissen T.H., Hayes B.J., Goddard M.E. (2001). Prediction of total genetic value using genome-wide dense marker maps. Genetics.

[33] Usai M.G., Goddard M.E., Hayes B.J. (2009). LASSO with cross-validation for genomic selection. Genet. Res..

[34] Habier D., Fernando R.L., Kizilkaya K., Garrick D.J. (2011). Extension of the bayesian alphabet for genomic selection. BMC Bioinform..

[35] Campos G.D.L., Naya H., Gianola D., Crossa J., Legarra A., Manfredi E., Weigel K., Cotes J.M. (2009). Predicting Quantitative Traits with Regression Models for Dense Molecular Markers and Pedigree. Genetics.

[36] Crain J., Mondal S., Rutkoski J., Singh R.P., Poland J. (2018). Combining High-Throughput Phenotyping and Genomic Information to Increase Prediction and Selection Accuracy in Wheat Breeding. Plant Genome.

[37] Holliday J.A., Wang T., Aitken S. (2012). Predicting Adaptive Phenotypes from Multilocus Genotypes in Sitka Spruce (*Picea sitchensis*) Using Random Forest. G3 Genes Genomes Genet..

[38] Long N., Gianola D., Rosa G.J.M., Weigel K.A. (2011). Application of support vector regression to genome-assisted prediction of quantitative traits. Theor. Appl. Genet..

[39] González-Camacho J.M., Campos G.D.L., Pérez P., Gianola D., Cairns J.E., Mahuku G., Babu R., Crossa J. (2012). Genome-enabled prediction of genetic values using radial basis function neural networks. Theor. Appl. Genet..

[40] González-Camacho J.M., Crossa J., Pérez-Rodríguez P., Ornella L., Gianola D. (2016). Genome-enabled prediction using probabilistic neural network classifiers. BMC Genom..

[41] Ma W., Qiu Z., Song J., Li J., Cheng Q., Zhai J., Ma C. (2018). A deep convolutional neural network approach for predicting phenotypes from genotypes. Planta.

[42] De Sousa I.C., Nascimento M., Silva G.N., Nascimento A.C.C., Cruz C.D., Silva F.F.E., De Almeida D.P., Pestana K.N., Azevedo C.F., Zambolim L. (2021). Genomic prediction of leaf rust resistance to Arabica coffee using machine learning algorithms. Sci. Agric..

[43] Zingaretti L.M., Gezan S.A., Ferrão L.F.V., Osorio L.F., Monfort A., Muñoz P.R., Whitaker V.M., Pérez-Enciso M. (2020). Exploring Deep Learning for Complex Trait Genomic Prediction in Polyploid Outcrossing Species. Front. Plant Sci..

[44] Sandhu K.S., Lozada D.N., Zhang Z., Pumphrey M.O., Carter A.H. (2021). Deep Learning for Predicting Complex Traits in Spring Wheat Breeding Program. Front. Plant Sci..

[45] Azodi C.B., Bolger E., McCarren A., Roantree M., Campos G.D.L., Shiu S.-H. (2019). Benchmarking Parametric and Machine Learning Models for Genomic Prediction of Complex Traits. G3 Genes Genomes Genet..

[46] Spindel J., Begum H., Akdemir D., Virk P., Collard B., Redoña E., Atlin G., Jannink J.-L., McCouch S.R. (2015). Genomic Selection and Association Mapping in Rice (*Oryza sativa*): Effect of Trait Genetic Architecture, Training Population Composition, Marker Number and Statistical Model on Accuracy of Rice Genomic Selection in Elite, Tropical Rice Breeding Lines. PLoS Genet..

[47] You Q., Yang X., Peng Z., Xu L., Wang J. (2018). Development and Applications of a High Throughput Genotyping Tool for Polyploid Crops: Single Nucleotide Polymorphism (SNP) Array. Front. Plant Sci..

[48] Korani W., Clevenger J., Chu Y., Ozias-Akins P. (2019). Machine Learning as an Effective Method for Identifying True Single Nucleotide Polymorphisms in Polyploid Plants. Plant Genome.

[49] Mochida K., Koda S., Inoue K., Nishii R. (2018). Statistical and Machine Learning Approaches to Predict Gene Regulatory Networks from Transcriptome Datasets. Front. Plant Sci..

[50] Huynh-Thu V.A., Irrthum A., Wehenkel L., Geurts P. (2010). Inferring Regulatory Networks from Expression Data Using Tree-Based Methods. PLoS ONE.

[51] Agarwal V., Shendure J. (2020). Predicting mRNA Abundance Directly from Genomic Sequence Using Deep Convolutional Neural Networks. Cell Rep..

[52] Beer M.A., Tavazoie S. (2004). Predicting Gene Expression from Sequence. Cell.

[53] Hafez D., Karabacak A., Krueger S., Hwang Y.-C., Wang L.-S., Zinzen R.P., Ohler U. (2017). McEnhancer: Predicting gene expression via semi-supervised assignment of enhancers to target genes. Genome Biol..

[54] Singh R., Lanchantin J., Robins G., Qi Y. (2016). DeepChrome: Deep-learning for predicting gene expression from histone modifications. Bioinformatics.

[55] Natarajan A., Yardımcı G.G., Sheffield N.C., Crawford G.E., Ohler U. (2012). Predicting cell-type–specific gene expression from regions of open chromatin. Genome Res..

[56] Washburn J.D., Mejia-Guerra M.K., Ramstein G., Kremling K.A., Valluru R., Buckler E.S., Wang H. (2019). Evolutionarily informed deep learning methods for predicting relative transcript abundance from DNA sequence. Proc. Natl. Acad. Sci. USA.

[57] Gardiner L.-J., Rusholme-Pilcher R., Colmer J., Rees H., Crescente J.M., Carrieri A.P., Duncan S., Pyzer-Knapp E.O., Krishna R., Hall A. (2021). Interpreting machine learning models to investigate circadian regulation and facilitate exploration of clock function. Genomics.

[58] Ramírez-González R.H., Borrill P., Lang D., Harrington S.A., Brinton J., Venturini L., Davey M., Jacobs J., van Ex F., Pasha A. (2018). The transcriptional landscape of polyploid wheat. Science.

[59] Gardiner L.-J., Joynson R., Omony J., Rusholme-Pilcher R., Olohan L., Lang D., Bai C., Hawkesford M., Salt D., Spannagl M. (2018). Hidden variation in polyploid wheat drives local adaptation. Genome Res..

[60] Gardiner L.-J., Spillane C., McKeown P. (2020). Understanding DNA Methylation Patterns in Wheat. Plant Epigenetics and Epigenomics.

[61] Perez-Riverol Y., Kuhn M., Vizcaíno J.A., Hitz M.-P., Audain E. (2017). Accurate and fast feature selection workflow for high-dimensional omics data. PLoS ONE.

[62] Li B., Zhang N., Wang Y.-G., George A.W., Reverter A., Li Y. (2018). Genomic Prediction of Breeding Values Using a Subset of SNPs Identified by Three Machine Learning Methods. Front. Genet..

[63] Degenhardt F., Seifert S., Szymczak S. (2019). Evaluation of variable selection methods for random forests and omics data sets. Brief. Bioinform..

[64] Haas R., Zelezniak A., Iacovacci J., Kamrad S., Townsend S., Ralser M. (2017). Designing and interpreting ‘multi-omic’ experiments that may change our understanding of biology. Curr. Opin. Syst. Biol..

[65] Vert J.-P., Lodhi H.M., Muggleton S.H. (2010). Reconstruction of Biological Networks by Supervised Machine Learning Approaches. Elements of Computational Systems Biology.

[66] Wang T., Wei S., Huang Z., Tang H., Zhang J., Ding Z., Huang K. (2020). MORONET: Multi-omics Integration via Graph Convolutional Networks for Biomedical Data Classification. bioRxiv.

[67] Xiaoxue L., Xuesong B., Longhe W., Bingyuan R., Shuhan L., Lin L. (2019). Review and Trend Analysis of Knowledge Graphs for Crop Pest and Diseases. IEEE Access.

[68] Timón-Reina S., Rincón M., Martínez-Tomás R. (2021). OUP accepted manuscript. Database.

[69] Dai X., Li J., Liu T., Zhao P.X. (2015). HRGRN: A Graph Search-Empowered Integrative Database of Arabidopsis Signaling Transduction, Metabolism and Gene Regulation Networks. Plant Cell Physiol..

[70] Venkatesan A., Ngompe G.T., El Hassouni N., Chentli I., Guignon V., Jonquet C., Ruiz M., Larmande P. (2018). Agronomic Linked Data (AgroLD): A knowledge-based system to enable integrative biology in agronomy. PLoS ONE.

[71] Hassani-Pak K., Singh A., Brandizi M., Hearnshaw J., Parsons J.D., Amberkar S., Phillips A.L., Doonan J.H., Rawlings C. (2021). KnetMiner: A comprehensive approach for supporting evidence-based gene discovery and complex trait analysis across species. Plant Biotechnol. J..

[72] Harfouche A.J., Jacobson D.A., Kainer D., Romero J.C., Harfouche A.H., Mugnozza G.S., Moshelion M., Tuskan G.A., Keurentjes J.J., Altman A. (2019). Accelerating Climate Resilient Plant Breeding by Applying Next-Generation Artificial Intelligence. Trends Biotechnol..

[73] Ghosal S., Blystone D., Singh A.K., Ganapathysubramanian B., Singh A., Sarkar S. (2018). An explainable deep machine vision framework for plant stress phenotyping. Proc. Natl. Acad. Sci. USA.

[74] Newman S.J., Furbank R.T. (2021). Explainable machine learning models of major crop traits from satellite-monitored continent-wide field trial data. Nat. Plants.

